# Effectiveness and Adherence of Nutritional Management via Electronic Patient-Reported Outcomes Platform in Patients With Cancer: Multicenter Prospective Longitudinal Cohort Study

**DOI:** 10.2196/75633

**Published:** 2025-11-28

**Authors:** Jiaxin Huang, Si-Wei Xie, Le Tian, Hui-Min Qu, Xi Zhang, Zhi-Gang Feng, Xin-Yi Wang, Zhen-Guang Du, Ming-Hui Zhang, Shu-Qing Wei, Jun Li, Li-Li Hong, Zhi-Cheng Zhou, Wen-Hui Yang, Wen-Hao Hu, Qian-Tong Dong, Ning Li, Min Yang, Meng Tang, Chen-Xin Song, Bao-Hua Zou, Sheng-Ling Qin, Rong Qin, Minghua Cong

**Affiliations:** 1 Comprehensive Oncology Department, National Cancer Center/National Clinical Research Center for Cancer/Cancer Hospital, Chinese Academy of Medical Sciences and Peking Union Medical College, No. 17, Panjiayuan Nanli, Chaoyang District, Beijing, 100021, China, 86 15001048699; 2 Department of Pediatrics, Johns Hopkins School of Medicine, Baltimore, Maryland, United States; 3 Department of Medicine, Gesi (Hebei) Medical Research Institute, Langfang, China; 4 Department of Interventional Medicine, Liaoning Provincial People's Hospital, Liaoning, China; 5 Department of Oncology, Chifeng Municipal Hospital, Chifeng, China; 6 Department of General Internal Medicine, Shanxi Province Cancer Hospital/Shanxi Hospital Affiliated to Cancer Hospital, Academy of Medical Sciences/Cancer Hospital Affiliated to Shanxi Medical University, Taiyuan, Shanxi, China; 7 Radiotherapy Department and Nutrition Department, Mianyang Cancer Hospital, Mianyang, China; 8 Department of Oncology, Tianjin Huanghe Hospital, Tianjin, China; 9 Department of General Surgery, First Affiliated Hospital of Wenzhou Medical University, Wenzhou, China

**Keywords:** nutritional management, electronic patient-reported outcomes platform, cancer, malnutrition, patient adherence

## Abstract

**Background:**

Personalized nutritional management during cancer remains challenging in clinical practice. The development of an electronic patient-reported outcome platform (ePROM) provides novel opportunities.

**Objective:**

This study aimed to evaluate the effectiveness and adherence of nutritional management using ePROM in patients with cancer.

**Methods:**

This multicenter prospective longitudinal cohort study included 6124 patients diagnosed with cancer. Exposure was defined as adherence to the ePROM journal, measured by the longest consecutive month of weekly entries. Dietary intake was reported via food selection, voice input, or meal photos. The primary outcomes were adequate energy intake (EI, ≥25 kcal/kg/day) and protein intake (PI, ≥1 g/kg/day), defined according to European Society for Clinical Nutrition and Metabolism (ESPEN) guidelines. Logistic regression analysis was conducted to identify the factors associated with EI and PI, reporting odds ratios (ORs) and 95% CIs. A restricted cubic spline plot was used to illustrate the association between the adjusted ORs and adherence duration. The semMediation approach was applied to assess the impact of multiple mediators on the outcomes.

**Results:**

The study cohort comprised 3741/6124 (61.1%) men and 2383 (38.9%) women, with a median age of 60.85 (IQR 53.3-68.3) years. Overall, 1024/6124 (16.7%) and 2591/6124 (42.3%) patients achieved adequate EI and PI scores, respectively. At one month, 499/1024 patients (48.7%) in the adequate EI group and 1287/2591 (49.7%) in the adequate PI group continued journaling, compared with 1879/5100 (36.8%) and 1091/3533 (30.9%) in the corresponding inadequate groups (*P*<.001). This trend remained significant in the second, third, and sixth months. Logistic regression analysis demonstrated that longer adherence to ePROM journaling was independently associated with adequate EI (OR 1.05, 95% CI 1.01‐1.08; *P*=.01) and PI (OR 1.22, 95% CI 1.16‐1.28; *P*<.001) after adjusting for confounders. Mediation analysis revealed that most symptoms did not significantly mediate these effects, except for constipation, reflux, and delirium, which showed statistical significance but minimal indirect effects.

**Conclusions:**

Nutritional management via ePROM is a feasible approach, with improved effectiveness as adherence duration increases. The observed benefits resulted primarily from direct effects rather than from symptom improvement.

## Introduction

Malnutrition is a common complication in patients with cancer that negatively affects clinical outcomes [1]. It is associated with increased treatment-related adverse effects, reduced therapeutic efficacy, impaired quality of life (QoL), and poor survival [2]. Given these detrimental effects, early recognition and timely intervention of malnutrition are key in cancer care. Evidence indicates that early nutritional therapy can enhance nutritional status, improve QoL, and augment responses to cancer treatment [3,4]. However, the effect of nutritional interventions on long-term outcomes such as survival remains inconclusive. One potential explanation for this is the relatively short duration of most nutritional interventions, which typically coincides with a single modality of anticancer treatment, lasting approximately 2‐6 months [5]. However, patients with cancer often undergo prolonged and recurrent treatment regimens, including chemotherapy, radiotherapy, surgery, and recovery. Malnutrition often develops, persists, or worsens throughout this trajectory and is influenced by dynamic factors, such as demographic characteristics, comorbidities, tumor type and location, tumor, node, metastasis (TNM) staging, and the nature of anticancer therapies [6]. To improve long-term clinical outcomes, nutritional interventions, including regular screening, comprehensive assessments, timely interventions, and continuous monitoring, should be sustained through cancer treatment. However, applying such a continuous, adaptive nutritional strategy within the traditional framework is challenging because of the lack of tools for prescribing dynamic nutritional regimens and ensuring continuous monitoring, regardless of whether patients are hospitalized or at home.

Despite continuous nutritional management, patient compliance remains a significant challenge. Capra et al [7], proposed that nutritional intervention consists of 2 key components: prescription and implementation. Successful intervention requires active participation from both the medical team and patients. The medical team can merely prescribe, and the patients are expected to implement the recommendations with good compliance. However, traditional approaches are constrained by several factors, including irregular clinic visits, loss of contact during outpatient periods, and delayed responses to patients’ dynamic nutritional needs [8]. A systematic review and meta-analysis revealed that compliance in randomized clinical trials involving dietary counseling with or without oral nutritional supplementation was generally poor, with often unmet energy and protein goals [9]. Moreover, poor patient compliance has been a major contributor to negative findings in several nutritional studies [10,11]. Therefore, nutritional interventions should provide timely and consistent access for patients to improve their outcomes.

At this time, telemedicine or remote monitoring is a means of overcoming these limitations and providing patient-centered continuous nutritional management. The electronic patient-reported outcome platform (ePROM) is a newly developed tool widely applied to monitor patient-reported symptoms in clinical cancer practice [12-16]. Accumulating evidence has confirmed its positive role in improving clinical outcomes by enabling better symptom reporting and communication through remote symptom self-reporting, promoting proactive management of symptoms through real-time clinician feedback, and facilitating clinician-patient interactions [17]. However, the evidence supporting the effectiveness of ePROM in nutritional management remains limited. Therefore, we conducted a prospective cohort study and developed an ePROM with three interconnected interfaces for patients, dieticians, and clinicians. The participants used the platform to report their nutritional status and symptoms while receiving timely feedback and adaptive nutritional regimens from dietitians and clinicians. This study aimed to evaluate the impact of ePROM-based nutritional management on achieving adequate energy and protein intake and reducing malnutrition risk in patients with cancer.

## Methods

### Study Participants and Design

**Figure 1.**
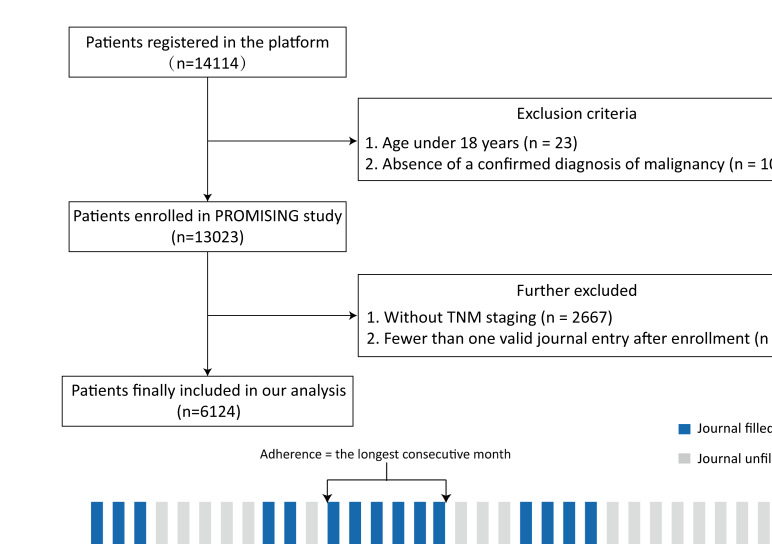
Flowchart of our study. PROMISING: patient-reported outcome management including surveillance and intervention in the nutritional group. TNM: tumor,node,metastasis;

Patient-Reported Outcome Management Including Surveillance and Intervention in Nutritional Group (PROMISING) is a multicenter prospective cohort study aimed at investigating the effect of patient-reported outcome (PRO)-based nutritional management across the disease continuum on clinical outcomes in patients with cancer (registration number: ChiCTR2100047535). Between March 2021 and April 2024, potentially eligible patients were consecutively recruited from oncology outpatient clinics and inpatient wards at 51 participating hospitals across China (Table S1 in Multimedia Appendix 1). During routine consultations, treating oncologists identified patients who met the preliminary eligibility criteria and referred them to the study platform. Trained research staff then screened patients against the full inclusion and exclusion criteria, explained the study procedures in detail, obtained written informed consent, and finally enrolled eligible patients in person, with enrollment simultaneously recorded in the ePROM system. Inclusion criteria were: (1) aged ≥18 years; (2) cytologically or histologically proven malignant tumors; and (3) an estimated life expectancy ≥3 months. Exclusion criteria included: (1) significant cardiovascular, renal, or hepatic comorbidities; (2) pregnancy or lactation; (3) severe mental disorders with poor compliance; (4) abnormal glucose metabolism (glycated hemoglobin ≥7.0%); (5) allergic to the contents of oral nutritional supplements; (6) inability to feed by mouth due to dysphagia or recurrent laryngeal nerve injury; and (7) deemed unsuitable for inclusion by investigators. The present study included patients from the PROMISING study who completed at least one eligible journal entry after enrollment (Figure 1).

### Description of ePROM

The ePROM used in this study was the PRO tool, especially for patients with cancer undergoing treatment (SHCD-PROTEC), developed by Shanghai Chudong Intelligent Technology Co, Ltd embedded within WeChat (Tencent Holdings Ltd)—the most widely used social media platform in China—it provided multiend access for physicians, dietitians, patients, and caregivers. Physicians could review patient information, including medical history, treatment regimens, and efficacy evaluations. Dietitians used the platform for nutritional screening, assessment, and individualized care planning, while patients and caregivers accessed nutrition plans and reported rehabilitation data. To enhance management effectiveness, improve adherence, and ensure data quality, the platform included three core functions: (1) message board: a chat-style feature enabling real-time communication among patients, caregivers, and health care providers; (2) real-time alerts: automated notifications to clinicians when patient-reported data exceeded predefined thresholds or showed abnormal fluctuations, prompting timely intervention; and (3) task reminders: automated prompts for missed journal entries, with persistent noncompliance triggering follow-up by dietitians to reassess the patient’s nutritional status.

### Data Collection and Variables Definition

Baseline characteristics, including demographic information (age, sex, and residence), comorbidities, and tumor-related data were collected upon patient enrollment. The study enrolled patients with tumors in the following subsites: head and neck, lung, breast, upper gastrointestinal, hepatobiliary and pancreatic, colorectal, urogenital, hematological, and others. Pathological staging was performed according to the eighth edition of the American Joint Committee on Cancer TNM staging system [18]. Information regarding anticancer treatments, including surgery, chemotherapy, radiotherapy, immunotherapy, and targeted therapy, was recorded for all patients to assess their treatment regimens. Nutritional risk screening and malnutrition assessment were performed by trained dietitians recruited specifically for this study within 48 hours of enrollment and repeated every 21‐28 days, using the electronic versions of the Nutritional Risk Screening 2002 (NRS-2002) and the Patient-Generated Subjective Global Assessment (PG-SGA) integrated into the ePROM system. All patients received standard nutrition support according to the European Society for Clinical Nutrition and Metabolism (ESPEN) Clinical Nutrition in Cancer guideline. All participants were required to complete at least one journal per week via their personal account. Each entry included dietary intake, which could be reported by selecting foods from the system’s database, using voice input, or uploading meal photographs. In addition, participants rated their symptoms on a 0‐10 scale. One adherent month was defined as four consecutive weeks during which all required weekly journals were completed. The adherence duration was calculated as the longest continuous number of such adherent months achieved by each participant. The ePROM management period was defined as the period from each participant’s enrollment date to the date of their last completed ePROM journal entry or the study analysis cutoff date, whichever occurred first.

### Outcomes

The primary outcomes were adequate energy intake (EI) and protein intake (PI) in patients with cancer. According to the ESPEN guidelines, the recommended thresholds for patients with cancer are an EI of at least 25.0 kcal/kg/day and a PI of at least 1.0 g/kg/day [2]. In our study, the EI and PI for each day were automatically calculated using the SHCD-PROTECT dietary analysis software based on the patients’ meal consumption and nutrient supplementation. Patients whose average EI and PI values met or exceeded these thresholds during the ePROM management period were classified as having adequate EI or PI values. The secondary outcome was malnutrition risk assessed using the NRS 2002. For the current analysis, we used the most recent NRS 2002 scores from the patients’ last recorded assessment to evaluate malnutrition risk.

### Statistical Analysis

All analyses were performed using the R software (version 4.3.2; R Project for Statistical Computing). Missing weeks of journal data were not imputed because journal completion was voluntary and reflected real-world behavior. To reduce the potential influence of irregular reporting, EI and PI were calculated as the mean of all available valid entries within each participant’s ePROM management period. Implausible total energy values (<50 kcal or >5000 kcal), together with their corresponding protein values, were excluded as outliers. Continuous variables are expressed as median (IQR) and compared using the Mann–Whitney *U* test, given that most variables did not meet normality assumptions. Categorical variables are expressed as absolute numbers or percentages and compared using the *χ^2^* test or Fisher exact test. Logistic regression analysis was performed to investigate risk factor-associated binary variables, reporting odds ratios (ORs) and 95% CIs. A restricted cubic spline plot was used to describe the potential nonlinear association between adjusted ORs and exposure. The impact of multiple mediators on the outcome was assessed using a semimediated approach, allowing for the estimation of direct, indirect, and total effects. To verify sample-size adequacy, a detectable-effect analysis for logistic regression was performed, assuming *α*=.05% and 80% power. Using the observed sample size (n=6124), event rates (EI=16.7% and PI=42.3%), and the SD of adherence (1.82), the minimum detectable OR was approximately 1.05 for EI and 1.04 for PI per 1-SD increase in adherence. Model stability was further confirmed using the events-per-variable criterion, with events-per-variable values of about 79 for EI and 199 for PI, both exceeding the conventional threshold of 10 events per variable. Statistical significance was defined as a 2-tailed *P* value of <.05.

### Ethical Considerations

This study was reviewed and approved by the Ethics Committee of the National Cancer Center, National Clinical Research Center for Cancer, Cancer Hospital, Chinese Academy of Medical Sciences and Peking Union Medical College, Beijing, China (approval number: 21/297‐2968), as well as the ethics committees of all participating institutions. All procedures involving human participants were conducted in accordance with the ethical standards of the institutional or national research committees and with the principles of the Declaration of Helsinki. Written informed consent was obtained from all participants prior to enrollment, covering both primary data collection and secondary analyses. All study data were stored in a secure, password-protected database. Identifiable patient information was removed and replaced with coded identifiers to protect privacy and confidentiality. Only authorized research personnel have access to the data. No financial or material compensation was provided to participants for their involvement in the study.

## Results

### Baseline Characteristics of Patients

A total of 14,114 patients were registered in the SHCD-PROTEC platform. After excluding 23 patients aged under 18 years and 1068 without a confirmed diagnosis of malignancy, 13,023 patients were enrolled in the PROMISING study. Among these, 2667 patients without TNM staging and 4232 with fewer than one valid journal entry after enrollment were further excluded. Consequently, 6124 patients were included in the final analysis, with a median age of 60.9 (53.3‐68.3) years. The cohort comprised 3741/6124 (61.1%) men and 2384/6124 (38.9%) women. The most common tumor sites were colorectal (1387/6124, 22.6%), lung (1346/6124, 22%), upper gastrointestinal (1311/6124, 21.4%), head and neck (615/6124, 10%), urogenital (595/6124, 9.7%), hepatobiliary and pancreatic (367/6124, 6%), and breast (293/6124, 4.8%) tumors. Approximately 80% (4882/6124) of the patients are diagnosed at an advanced stage. The median duration of the ePROM management period was 89 (32.8‐172) days. During the entire period, 1024/6124 (16.7%) patients achieved adequate EI, and 2591/6124 (42.3%) met the required PI. Patients who did not meet their goals tended to be older, male, in advanced stages of cancer, undergoing anticancer treatment, or with comorbidities (diabetes and hypertension). Detailed baseline information is provided in Table 1.

**Table 1.** Baseline profile of patients.

Variables	All participants (N=6124)	Average calorie intake, kcal/kg/day			Average protein intake, g/kg/day		
		Inadequate (n=5100)	Adequate (n=1024)	*P* value	Inadequate (n=3533)	Adequate (n=2591)	*P* value
Age (years), median (IQR)	60.9 (53.3‐68.3)	61.33 (53.6‐68.6)	59.2 (51.3‐66.8)	<.001	61.4 (53.7‐68.5)	60.3 (52.7‐68.0)	.01
Sex, n (%)				<.001			<.001
Male	3740 (61.1)	3225 (63.2)	515 (50.3)		2249 (63.7)	1491 (57.5)	
Female	2384 (38.9)	1875 (36.8)	509 (49.7)		1284 (36.3)	110 (42.5)	
Residency, n (%)				.13			<.001
Rural or Township	4813 (78.6)	4027 (79)	786 (76.8)		2869 (81.2)	1744 (75)	
Urban or City	1311 (21.4)	1073 (21)	238 (23.2)		664 (18.8)	647 (25)	
Tumor site, n (%)				<.001			<.001
Head and neck	615 (10)	486 (9.5)	129 (12.6)		344 (9.7)	271 (10.5)	
Lung	1346 (22)	1182 (23.2)	164 (16.0)		813 (23)	533 (20.6)	
Breast	293 (4.8)	232 (4.5)	61 (6.0)		176 (5)	117 (4.5)	
Upper gastrointestinal	1311 (21.4)	1077 (21.1)	234 (22.9)		705 (20)	606 (23.4)	
Hepatobiliary and pancreatic	367 (6)	303 (5.9)	64 (6.2)		189 (5.3)	178 (6.9)	
Colorectal	1387 (22.6)	1205 (23.6)	182 (17.8)		883 (25)	504 (19.5)	
Urogenital	595 (9.7)	438 (8.6)	157 (15.3)		296 (8.4)	299 (11.5)	
Hematological	81 (1.3)	67 (1.3)	14 (1.4)		45 (1.3)	36 (1.4)	
Others	129 (2.1)	110 (2.2)	19 (1.9)		82 (2.3)	47 (1.8)	
TNM^a^ stage, n (%)				<.001			<.001
Early (I and II)	1242 (20.3)	1001 (19.6)	241 (23.5)		700 (19.8)	542 (20.9)	
Advanced (III and IV)	4882 (79.7)	4099 (80.4)	783 (76.5)		2833 (80.2)	2049 (79.1)	
Anticancer treatments, n (%)							
Chemotherapy				<.001			<.001
Yes	4162 (68)	3414 (66.9)	748 (73)		2285 (64.7)	1877 (72.4)	
No	1962 (32)	1686 (33.1)	276 (27)		1248 (35.3)	714 (27.6)	
Radiotherapy				<.001			<.001
Yes	1986 (32.4)	1533 (30.1)	453 (44.2)		1052 (29.8)	934 (36)	
No	4138 (67.6)	3567 (69.9)	571 (55.8)		2481 (70.2)	1657 (64)	
Surgery				.07			.33
Yes	2880 (47)	2371 (46.5)	509 (49.7)		1642 (46.5)	1238 (47.8)	
No	3244 (53)	2729 (53.5)	515 (50.3)		1891 (53.5)	1353 (52.2)	
Immunotherapy				.09			.005
Yes	1722 (28.1)	1411 (27.7)	311 (30.4)		944 (26.7)	778 (30)	
No	4402 (71.9)	3689 (72.3)	713 (69.6)		2589 (73.3)	1813 (70)	
Targeted therapy		1631/3469 (32.0/68.0)	362/662 (35.4/64.6)	.04		887/1704 (34.2/65.8)	.02
Yes	1993 (32.5)	1631 (32)	362 (35.4)		1106 (31.3)	887 (34.2)	
No	4131 (67.5)	3469 (68)	662 (64.6)		2427 (68.7)	1704 (65.8)	
Comorbidities, n (%)							
Diabetes				<.001		251/2340 (9.7/90.3)	.02
Yes	660 (10.8)	587 (11.5)	73 (7.1)		409 (11.6)	251 (9.7)	
No	5464 (89.2)	4513 (88.5)	951 (92.9)		3124 (88.4)	2340 (90.3)	
Hypertension				<.001			<.001
Yes	1232 (20.1)	1109 (21.7)	123 (12)		807 (22.8)	425 (16.4)	
No	4892 (79.9)	3991 (78.3)	901 (88)		2726 (77.2)	2166 (83.6)	
Period of ePROM^b^ management, days	89 (32.8‐172)	86.0 (31‐165)	110 (41‐227)	<.001	74.0 (26‐152)	115 (46‐211.5)	<.001
Adherence duration for ePROM journal							
≥1 month	2378 (38.38)	1879 (36.8)	499 (48.7)	<.001	1091 (30.9)	1287 (49.7)	<.001
≥2 months	783 (12.8)	580 (11.4)	203 (19.8)	<.001	251 (7.1)	532 (20.5)	<.001
≥3 months	393 (6.4)	287 (5.6)	106 (10.4)	<.001	115 (3.3)	278 (10.7)	<.001
≥6 months	107 (1.7)	80 (1.6)	27 (2.6)	.02	3 (0.9)	76 (2.9)	<.001

a TNM: tumor, node, metastasis.

b ePROM: electronic patient-reported outcomes platform.

### Association Between Adherence for ePROM Journal With Adequate EI and PI

**Figure 2.**
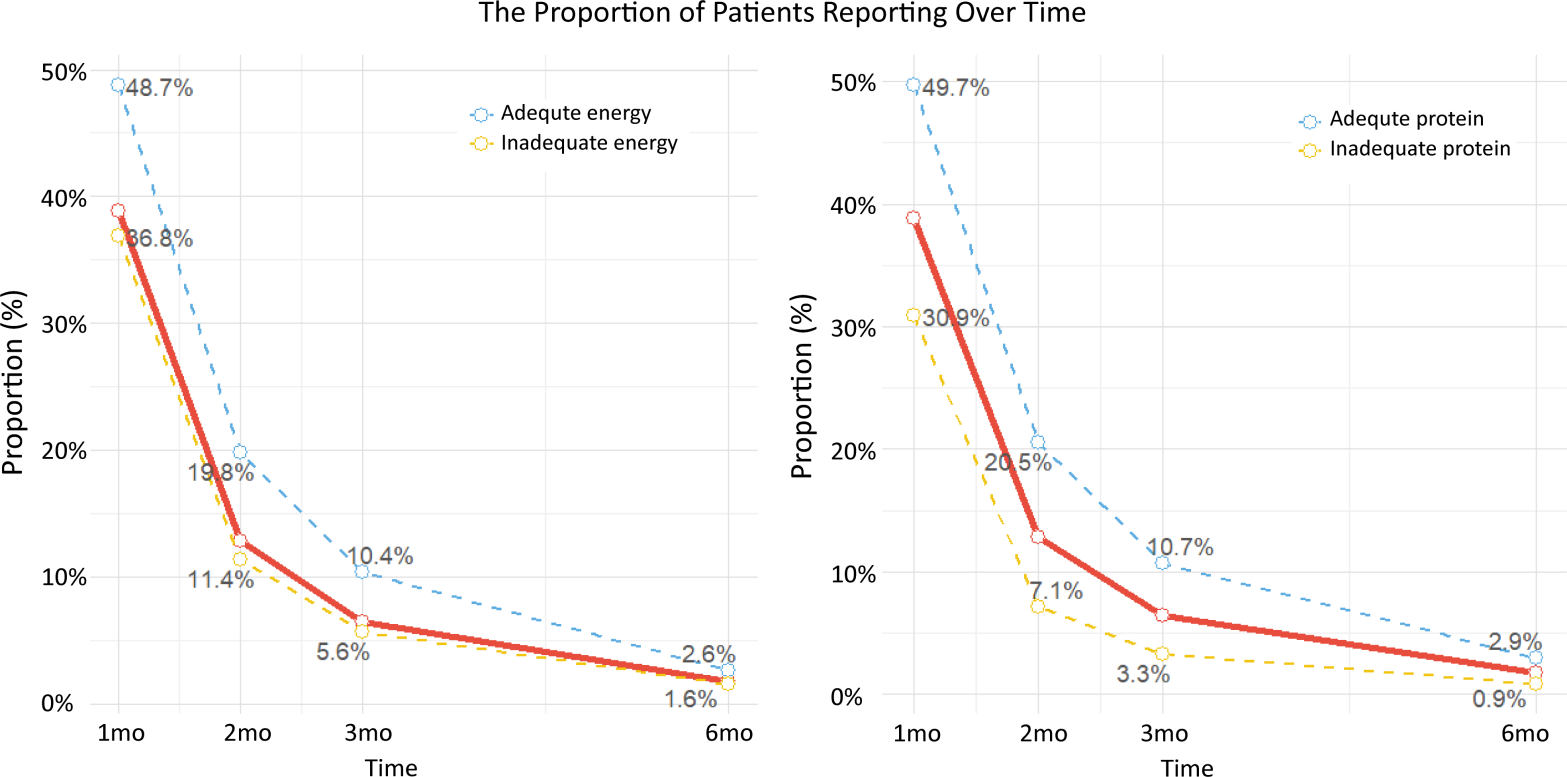
The proportion of patients reporting with time.

**Figure 3.**
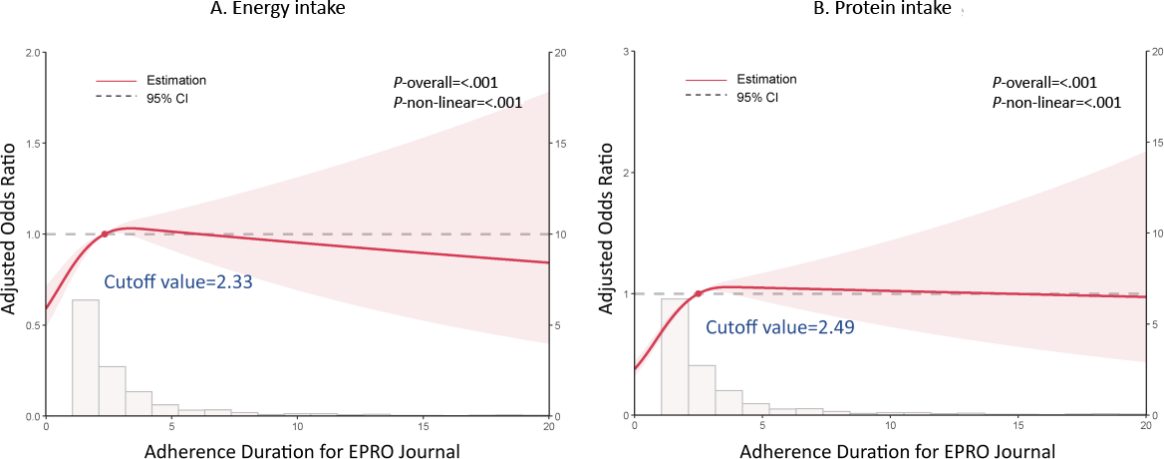
Restrict cubic spline plots for ePROM Journal adherence with adequate energy and protein intake.

As shown in Table 1 and Figure 2, patients with adequate EI and PI demonstrated significantly higher adherence to completing the ePROM journals. In the adequate EI group, 499/1024 patients (48.7%) continued to fill out the journals for one month, compared to 1879/5100 patients (36.8%) in the inadequate EI group (*P*<.001). Similarly, in the adequate PI group, 1287/2591 patients (49.7%) adhered for one month, compared to 1091/3533 patients (30.9%) in the inadequate PI group (*P*<.001). After 2 months, adherence remained higher in the adequate groups: 203/1024 patients (19.8%) versus 580/5100 (11.4%) in the EI groups, and 532/2591 (20.5%) versus 251/3533 (7.1%) in the PI groups (all *P*<.001). A similar significant trend was observed at 3 and 6 months. Logistic analysis revealed that long adherence duration for ePRO journals served as an independent protective factor associated with both EI (OR 1.05, 95% CI 1.01‐1.08; *P*=.01) and PI (OR 1.22, 95% CI 1.16‐1.28; *P*<.001), after adjusting for age, sex, residency, tumor site, TNM stage, anticancer treatments, diabetes, hypertension, and ePROM management period (Table 2). When we categorized the adherence duration into 1, 3, and 6 months, we observed that the adjusted OR increased as the adherence duration increased. The restricted cubic spline plot described an L-shape relationship between adherence duration for ePROM the journal with adjusted OR, with the cut-off value of 2.33 months for EI and 2.49 months for PI (Figure 3A-B).

**Table 2.** Logistic regression analysis between ePROM journal adherence with adequate energy/protein intake.

Variables	Adequate energy intake				Adequate protein intake			
	Univariate		Multivariate		Univariate		Multivariate	
	OR^a^ (95% CI)	*P* value	OR (95% CI)	*P* value	OR (95% CI)	*P* value	OR (95% CI)	*P* value
Adherence duration for ePROM^b^ journal (months)	1.09 (1.06‐1.13)	<.001	1.05 (1.01‐1.08)	.01	1.33 (1.27‐1.39)	<.001	1.22 (1.16‐1.28)	<.001
≥1 month	1.63 (1.42‐1.87)	<.001	1.42 (1.23‐1.64)	<.001	2.21 (1.99‐2.45)	<.001	1.86 (1.66‐2.08)	<.001
≥2 months	1.93 (1.61‐2.29)	<.001	1.61 (1.33‐1.96)	<.001	3.38 (2.88‐3.97)	<.001	2.57 (2.17‐3.05)	<.001
≥3 months	1.94 (1.53‐2.44)	<.001	1.48 (1.14‐1.91)	.003	3.57 (2.86‐4.48)	<.001	2.37 (1.87‐3.01)	<.001

a OR: odds ratio.

b ePROM: electronic patient-reported outcomes platform.

### Mediation Analysis of Symptoms

Across the study, appetite loss was the most prevalent symptom, reported in 5089/6124 patients (83.1%), followed by fatigue in 2259 (36.9%), dry mouth in 1388 (22.7%), pain in 1237 (20.2%), nausea in 822 (13.4%), constipation in 732 (12%), anxiety in 730 (11.9%), abdominal bloating in 654 (10.7%), cough in 624 (10.2%), diarrhea in 350 (5.7%), depression in 335 (5.5%), dyspnea in 218 (3.6%), delirium in 79 (1.3%), and reflux in 59 (1%) (Figure S1 in Multimedia Appendix 1). The median appetite score was 5.83 (IQR 3.50‐7.00), while fatigue had a median score of 0.33 (IQR 0.00‐1.67). More detailed symptom score data across patient groups are presented in Table S2 in Multimedia Appendix 1. Subgroup analysis revealed variations in the positive effects of long-term ePROM adherence across different symptom groups (Figure S1 in Multimedia Appendix 1). A significant interaction effect was observed for fatigue, appetite, abdominal bloating, diarrhea, and reflux, suggesting that ePROM adherence had different effects depending on the presence of symptoms. Furthermore, a mediation analysis was conducted to assess potential indirect effects (Table S3 in Multimedia Appendix 1). . The results indicated that no significant mediation effects were observed for most symptoms. Although constipation, reflux, and delirium were significant, the proportion of indirect effects was minimal.

### Logistics Regression Analysis for Malnutrition Risk

Among the total cohort of 4978 participants, data from the most recent NRS 2002 scores recorded in the patients’ most recent journals were available. Of these, 2135 patients with cancer were found to be at risk of malnutrition. Univariate logistic regression analysis revealed that several factors, including age, sex, tumor site, TNM stage, chemotherapy history, hypertension, the period of ePROM management, and adherence to ePROM journals, were significantly associated with malnutrition risk. Subsequent multivariate logistic regression analysis confirmed that a longer period of ePRO management was an independent and favorable factor that reduced the risk of malnutrition (Table 3).

**Table 3.** Logistics analysis between electronic patient-reported outcome platform journal adherence with Nutrition Risk Screening 2002.

Variables	Univariate		Multivariate	
	OR^a^ (95% CI)	*P* value	OR (95% CI)	*P* value
Age (years)	1.02 (1.01‐1.02)	<.001	1.02 (1.01‐1.03)	<.001
Sex				
Female	Reference			
Male	1.21 (1.08‐1.36)	.001	0.91 (0.79‐1.04)	.15
Residency				
Rural or Township	Reference		—	—
Urban or City	1.09 (0.96‐1.25)	.18	—	—
Tumor site				
Head and Neck	Reference			
Lung	0.47 (0.37‐0.58)	<.001	0.39 (0.31‐0.49)	<.001
Breast	0.31 (0.22‐0.44)	<.001	0.31 (0.22‐0.45)	<.001
Upper Gastrointestinal	1.16 (0.92‐1.45)	.21	1.05 (0.83‐1.34)	.67
Hepatobiliary and Pancreatic	0.87 (0.65‐1.17)	.36	0.75 (0.55‐1.01)	.06
Colorectal	0.57 (0.46‐0.71)	<.001	0.52 (0.41‐0.66)	<.001
Urogenital	0.48 (0.37‐0.63)	<.001	0.47 (0.35‐0.62)	<.001
Hematological	0.80 (0.48‐1.32)	.39	0.78 (0.46‐1.32)	.35
Others	0.40‐0.25-0.61)	<.001	0.34 (0.21‐0.53)	<.001
TNM^b^ stage				
Early (I and II)	Reference			
Advanced (III and IV)	1.25 (1.08‐1.43)	.002	1.15 (1.07‐1.23)	<.001
Anticancer treatments (Yes vs No)				
Chemotherapy	0.79 (0.70‐0.89)	<.001	0.75 (0.66‐0.86)	<.001
Radiotherapy	1.08 (0.96‐1.21)	.22	—	—
Surgery	0.98 (0.88‐1.10)	.77	—	—
Immunotherapy	0.93 (0.82‐1.05)	.24	—	—
Targeted therapy	0.93 (0.83‐1.05)	.25	—	—
Comorbidities (Yes vs No)				
Diabetes	1.00 (0.83‐1.19)	.96		
Hypertension	0.80 (0.70‐0.93)	.003	0.72 (0.62‐0.83)	<.001
Period of ePROM^c^ management	0.998 (0.997‐0.998)	<.001	0.998 (0.997‐0.998)	<.001
Adherence duration for ePROM journal	0.96 (0.93‐0.99)	.02	1.02 (0.98‐1.05)	.36

a OR: odds ratio.

b TNM: tumor, node, metastasis.

c ePROM: electronic patient-reported outcomes platform.

## Discussion

### Principal Findings

Tailored and continuous nutritional management throughout the disease course is crucial for patients with cancer. However, traditional clinical care faces several barriers to implementing nutritional management, including time consumption, poor patient compliance, and high rates of follow-up loss [8,9]. The newly developed ePROM facilitates real-time PRO data collection, monitoring, and personalized feedback, thereby offering a promising solution for improving the effectiveness of nutritional management [12,13,15,16]. Our research team has previously developed an ePROM specifically designed to provide nutritional management to patients with cancer. The use of our platform is flexible, free, and convenient to ensure the continuity and personalization of nutritional management, as well as to reduce the workload of health care teams. As with traditional nutritional interventions, the success of this approach depends largely on patient adherence. In this study, fewer than 40% of patients consistently completed ePROM journals beyond one month, with adherence declining significantly over time. This trend was observed in both the adequate and inadequate EI and PI groups; however, patients in the adequate EI and PI group demonstrated significantly higher proportions of sustained ePROM use at > 1, 2, 3, and 6 months. Multivariate logistic regression analysis adjusted for potential confounders confirmed that prolonged ePROM adherence was an independent protective factor associated with adequate EI and PI. However, in relation to malnutrition risk, patients with a longer ePROM management period showed a significantly lower risk of malnutrition at the final follow-up, rather than those with higher adherence. Further studies are required to clarify whether the duration of ePROM management or the level of adherence exerts a greater influence on nutritional outcomes. Subgroup analysis revealed variations in the effects of nutritional management across different symptom groups. Therefore, to explore the intrinsic role of nutritional management via ePROM further, we performed a mediation analysis. The results demonstrated that the achievement of EI and PI goals was directly influenced by nutritional interventions via ePROM rather than by the improvement of symptoms.

### Comparison to Prior Work

The ePROM adherence rates in previous clinical trials range from 65% to > 90% [19,20]. Gebert et al [21] recently reported that the adherence to ePROM follow-up assessments in patients with invasive early breast cancer and ductal carcinoma in situ was moderate, ranging from 60% to 80%. In comparison, adherence in our prospective longitudinal study was relatively low, suggesting that many patients may still lack the capacity or motivation to actively engage in the self-management of their conditions. We suspect that this may be attributed to several potential reasons: (1) the importance of continuous nutritional monitoring and its benefit for health outcomes were not sufficiently addressed to patients, which may have contributed to low motivation to maintain long-term participation [22]. (2) Although our platform included a message-alert system to remind patients to record in their journals, it may not have been sufficient to ensure consistent engagement. Evidence suggests that patients prefer automatic telephone calls or video calls because these methods help them feel more closely connected with their caregivers [23]. (3) The process of regularly filling out journals using a digital platform may have been time-consuming or burdensome for some patients, especially the elderly and those with multiple advanced conditions. In contrast to the study by Gebert et al [21], most patients in our study were in advanced disease stages, which might have led to a lower adherence rate. (4) Unlike previous studies, nutritional management demands more frequent, prolonged, and detailed self-reporting, which may pose challenges for patients in consistently completing medical journals. Therefore, it is essential to explore the optimal balance between gathering sufficient information for prescribing nutritional regimens and minimizing the patient burden.

A study from Greece showed that meeting at least the minimum amounts of protein and energy recommended by ESPEN may prevent weight loss and improve nutritional status [24]. In our study, more than 15% and 40% of the patients ultimately achieved their goals for average EI and PI, respectively. The relatively high achievement rate of the PI may be attributed to the popular use of nutrient supplements, particularly protein supplements. This aligns with a previous randomized controlled parallel-group study, which confirmed that a high-protein oral nutritional supplement can enable the majority of patients with cancer to meet the PI recommendations [25]. Furthermore, our study showed that prolonged ePROM adherence was an independent protective factor associated with adequate EI and PI. During cancer, patients often experience a variety of moderate-to-severe symptom burdens that significantly impair their daily routine and QoL [26]. Recent research on ePROM has primarily focused on symptom monitoring, giving it the potential to effectively track symptom burden [15,27]. Nutritional treatment, especially counseling, is also aimed at symptom management [26]. In our study, the results demonstrated that the achievement of EI and PI goals was directly influenced by nutritional interventions via ePROM rather than by the improvement of symptoms, such as pain, fatigue, dyspnea, cough, loss of appetite, dry mouth, nausea, abdominal bloating, diarrhea, constipation, reflux, anxiety, depression, and delirium. This suggests a fundamental difference between our ePROM approach and those commonly used in clinical practice, which tend to focus on symptom management. However, further research is needed to determine whether a combined approach that addresses both nutritional and symptom management can lead to better clinical outcomes in patients with cancer.

### Limitations

This study had some limitations. First, the overall adherence rate to ePROM was relatively low, and the duration of ePROM management varied among participants despite statistical adjustment, which may have hindered the comprehensive evaluation of long-term clinical outcomes. In addition, defining the ePROM management period based on the date of the last completed journal entry, although applied uniformly across all participants, may have introduced some arbitrariness. Second, our study did not specify a fixed day for journal entry, which may have introduced bias, as patients were more likely to report symptoms when they experienced discomfort or dietary difficulties. Moreover, voluntary journal completion led to inevitable missing entries; using mean values of available data and excluding implausible outliers may still have introduced minor bias. Underreporting, overreporting, and entry errors are also inherent to food diaries and were not validated by dietitians in our study due to the self-reported, real-world nature. Third, the absence of a control group that received standard nutritional management limited the ability to directly compare the effects of conventional care. Fourth, both the exposure and outcomes were derived from the journal of ePROM, which may have introduced common source bias. Finally, the definition of adherence used in this study requires further validation, as no standardized criteria have yet been established in the digital health field. Large-scale randomized clinical trials with long-term follow-up are required to confirm the effectiveness of nutritional management using ePROM in patients with cancer.

### Conclusions

In conclusion, our study demonstrated the potential and feasibility of using ePROM for nutritional management. However, adherence remains a challenge in real-world prospective studies and must be addressed. Longer duration of adherence to the ePROM journal significantly improves the likelihood of achieving adequate EI and PI goals, though its association with malnutrition risk was not statistically significant. Furthermore, the effectiveness of our ePROM approach is directly attributed to nutritional management rather than symptom monitoring, which is commonly used in clinical care.

## Supplementary material

10.2196/75633 Multimedia Appendix 1 Supplementary figures and tables for subgroup analyses and other description.

10.2196/75633 Checklist 1 STROBE (Strengthening the Reporting of Observational Studies in Epidemiology) statement—checklist of items that should be included in reports of cohort studies.

## Abbreviations

EI: energy intake
ePROM: electronic patient-reported outcome platform
ESPEN: European Society for Clinical Nutrition and Metabolism
NRS 2002: Nutrition Risk Screening 2002
ORs: odds ratios
PG-SGA: Patient-Generated Subjective Global Assessment
PI: protein intake
PROMISING: patient-reported outcome management including surveillance and intervention in nutritional group
QoL: quality of life
TNM: tumor/node/metastasis
