# Supplementary material for: Effectiveness and Adherence of Nutritional Management via Electronic Patient-Reported Outcomes Platform in Patients With Cancer: Multicenter Prospective Longitudinal Cohort Study
Source: JMIR Cancer. 2025 Nov 28;11:e75633. doi: 10.2196/75633 (PMC12669918; doi:10.2196/75633)
Supplement: Multimedia Appendix 1 [file cancer-v11-e75633-s001.docx]

**Supplementary files**

**Figure S1 Subgroup Analyses Stratified by Symptoms**

**
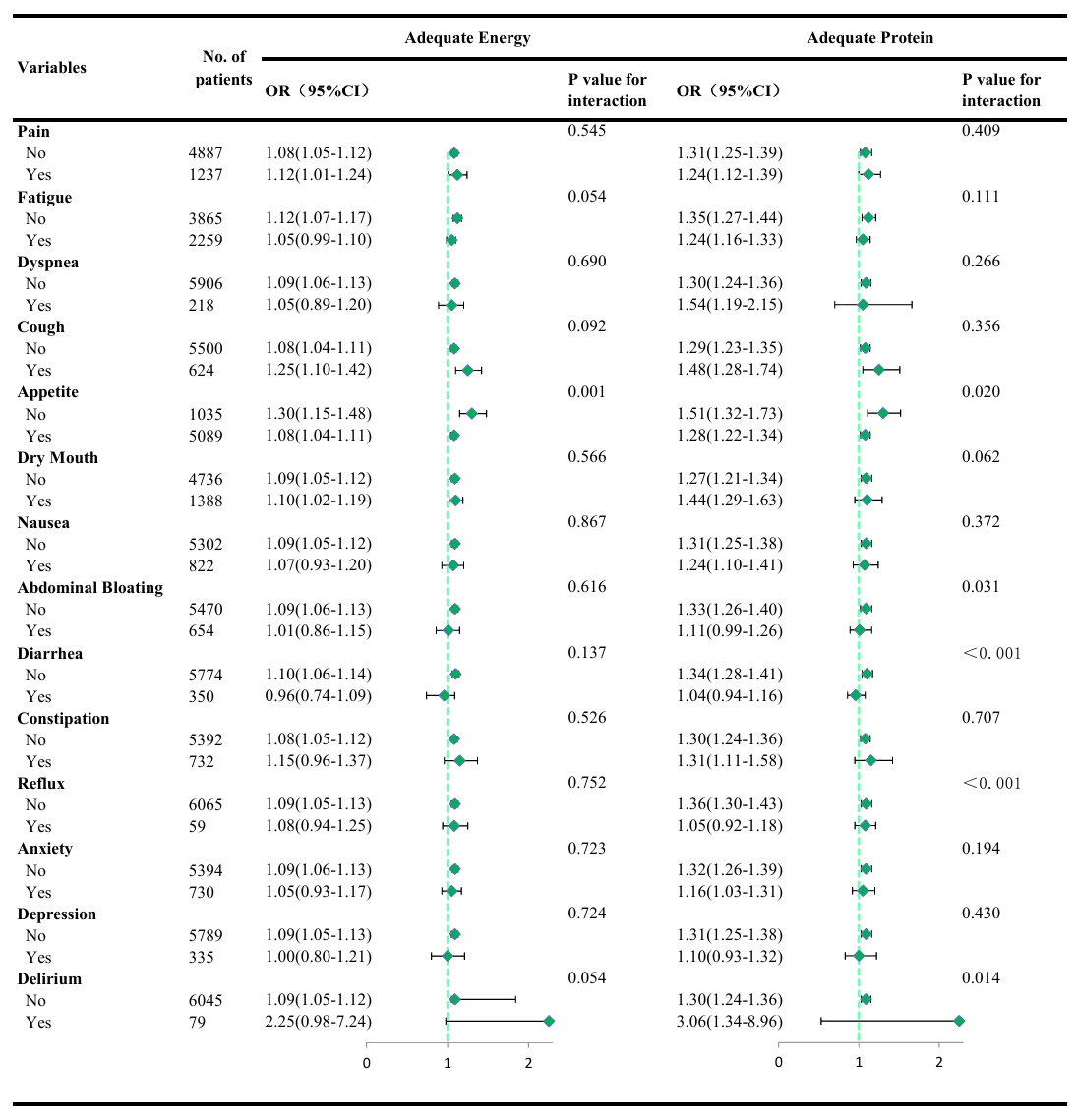
**

**Table S1 Participating Hospitals Across China and the Number of Patients Enrolled**

| Participating Hospital | Patients Enrolled |
| --- | --- |
| Nanfang Hospital, Southern Medical University | 593 |
| Northern Theater Command General Hospital | 455 |
| Shandong Cancer Hospital | 413 |
| The First Hospital of Shanxi Medical University | 352 |
| Mianyang Cancer Hospital | 347 |
| Cancer Hospital, Chinese Academy of Medical Sciences | 302 |
| Dujiangyan Medical Center | 300 |
| Harbin Medical University Cancer Hospital | 266 |
| Ordos Central Hospital | 264 |
| The First Hospital of Jilin University | 252 |
| Chongqing University Cancer Hospital | 234 |
| Chifeng Municipal Hospital | 217 |
| Sir Run Run Shaw Hospital, Zhejiang University School of Medicine | 210 |
| Weihai Municipal Hospital | 202 |
| Liaoning Cancer Hospital | 177 |
| Ningbo Hospital, Renji Hospital, Shanghai Jiao Tong University School of Medicine | 145 |
| Sichuan Cancer Hospital | 124 |
| Qinhuai Medical District, Eastern Theater Command General Hospital, Nanjing | 94 |
| Fujian Cancer Hospital | 92 |
| The Second Hospital of Tianjin Medical University | 92 |
| The First Affiliated Hospital of Wenzhou Medical University | 83 |
| Northern Jiangsu People’s Hospital | 82 |
| Tianjin Huanghe Hospital | 75 |
| The First Affiliated Hospital of Xi’an Jiaotong University | 74 |
| Xi’an International Medical Center Hospital | 72 |
| Yiwu Central Hospital | 67 |
| Qijiang District People’s Hospital of Chongqing | 56 |
| Beijing Luhe Hospital | 53 |
| Jiangsu Provincial People’s Hospital | 44 |
| Liaoning Provincial People’s Hospital | 43 |
| The First Affiliated Hospital of Chengdu Medical College | 38 |
| Kaifeng Central Hospital | 36 |
| Anhui Provincial Hospital | 30 |
| The First Affiliated Hospital of Jiamusi University | 27 |
| East Branch, Renji Hospital, Shanghai Jiao Tong University | 27 |
| Sanmenxia Central Hospital | 27 |
| Sichuan Provincial Construction Hospital | 24 |
| Three Gorges Hospital, Chongqing University | 23 |
| Meishan Traditional Chinese Medicine Hospital | 19 |
| Huaihe Hospital of Kaifeng | 16 |
| Tongji Hospital, Shanghai Tongji University | 15 |
| Zhejiang Cancer Hospital | 13 |
| Bethune Hospital of Shanxi | 13 |
| Guangxi Medical University Cancer Hospital | 9 |
| Shenyang Tenth People’s Hospital | 7 |
| Deyang Cancer Hospital | 6 |
| Mianyang Central Hospital | 5 |
| Xiamen Hong’ai Hospital | 5 |
| Xuzhou Central Hospital | 2 |
| West China Hospital, Sichuan University | 1 |
| Shanxi Provincial Cancer Hospital | 1 |

**Table S2 Comparison of Symptoms Scores Among Patients Group**

| Symptoms | All participants (N=6124) | Average calorie intake, kcal/kg/day | | | | | Average protein intake, g/kg/day | | | |
| --- | --- | --- | --- | --- | --- | --- | --- | --- | --- | --- |
|  |  | Inadequate (N=5100) | | Adequate (N=1024) | | *P* value | Inadequate (N=3533) | | Adequate (N=2591) | *P* value |
| Pain | 0.00(0.00-0.60) | 0.00(0.00-0.64) | | 0.00(0.00-0.50) | | .311 | 0.00(0.00-0.71) | | 0.00(0.00-0.50) | .21 |
| Fatigue | 0.33(0.00-1.57) | 0.33(0.00-1.67) | | 0.12(0.00-1.01) | | <.001 | 0.43(0.00-1.83) | | 0.20(0.00-1.33) | <.001 |
| Dyspnea | 0.00(0.00-0.00) | 0.00(0.00-0.00) | | 0.00(0.00-0.00) | | .021 | 0.00(0.00-0.00) | | 0.00(0.00-0.00) | .276 |
| Cough | 0.00(0.00-0.00) | 0.00(0.00-0.00) | | 0.00(0.00-0.07) | | .021 | 0.00(0.00-0.00) | | 0.00(0.00-0.00) | .160 |
| Appetite | 5.83(3.50-7.00) | 5.67(3.50-7.00) | | 6.50(4.32-7.80) | | <.001 | 5.50(3.25-6.88) | | 6.21(4.25-7.40) | <.001 |
| Dry Mouth | 0.00(0.00-0.72) | 0.00(0.00-0.75) | | 0.00(0.00-0.67) | | .761 | 0.00(0.00-0.88) | | 0.00(0.00-0.59) | .055 |
| Nausea | 0.00(0.00-0.25) | 0.00(0.00-0.27) | | 0.00(0.00-0.12) | | .003 | 0.00(0.00-0.33) | | 0.00(0.00-0.15) | .001 |
| Bloating | 0.00(0.00-0.06) | 0.00(0.00-0.00) | | 0.00(0.00-0.18) | | .002 | 0.00(0.00-0.00) | | 0.00(0.00-0.14) | <.001 |
| Diarrhea | 0.00(0.00-0.00) | 0.00(0.00-0.00) | | 0.00(0.00-0.00) | | .227 | 0.00(0.00-0.00) | | 0.00(0.00-0.00) | <.001 |
| Constipation | 0.00(0.00-0.12) | 0.00(0.00-0.12) | | 0.00(0.00-0.12) | | .852 | 0.00(0.00-0.22) | | 0.00(0.00-0.06) | .001 |
| Reflux | 0.00(0.00-0.00) | 0.00(0.00-0.00) | | 0.00(0.00-0.00) | | .366 | 0.00(0.00-0.00) | | 0.00(0.00-0.00) | .434 |
| Anxiety | 0.00(0.00-0.09) | 0.00(0.00-0.00) | | 0.00(0.00-0.33) | | <.001 | 0.00(0.00-0.00) | | 0.00(0.00-0.19) | <.001 |
| Depression | 0.00(0.00-0.00) | 0.00(0.00-0.00) | | 0.00(0.00-0.00) | | <.001 | 0.00(0.00-0.00) | | 0.00(0.00-0.00) | <.001 |
| Delirium | 0.00(0.00-0.00) | 0.00(0.00-0.00) | | 0.00(0.00-0.00) | | .221 | 0.00(0.00-0.00) | | 0.00(0.00-0.00) | .864 |
|  | Adherence duration for ePROM journal | | | | | | | | | |
|  | Less than one month  (N=3746) | | One month  (N=1595) | | Two months  (N=390) | | | Three months  (N=393) | | *P* value |
| Pain | 0.00(0.00-0.50) | | 0.00(0.00-0.75) | | 0.00(0.00-0.46) | | | 0.08(0.00-0.56) | | <.001 |
| Fatigue | 0.14(0.00-1.50) | | 0.38(0.00-1.57) | | 0.47(0.00-1.64) | | | 0.48(0.00-1.71) | | <.001 |
| Dyspnea | 0.00(0.00-0.00) | | 0.00(0.00-0.00) | | 0.00(0.00-0.00) | | | 0.00(0.00-0.00) | | <.001 |
| Cough | 0.00(0.00-0.00) | | 0.00(0.00-0.12) | | 0.00(0.00-0.08) | | | 0.00(0.00-0.25) | | <.001 |
| Appetite | 5.50(3.33-7.00) | | 6.13(4.25-7.22) | | 6.10(4.00-7.00) | | | 6.14(4.47-7.08) | | <.001 |
| Dry Mouth | 0.00(0.00-0.83) | | 0.00(0.00-0.67) | | 0.00(0.00-0.64) | | | 0.06(0.00-0.57) | | .008 |
| Nausea | 0.00(0.00-0.00) | | 0.00(0.00-0.30) | | 0.00(0.00-0.29) | | | 0.05(0.00-0.40) | | <.001 |
| Bloating | 0.00(0.00-0.00) | | 0.00(0.00-0.22) | | 0.00(0.00-0.30) | | | 0.00(0.00-0.25) | | <.001 |
| Diarrhea | 0.00(0.00-0.00) | | 0.00(0.00-0.00) | | 0.00(0.00-0.14) | | | 0.00(0.00-0.12) | | <.001 |
| Constipation | 0.00(0.00-0.00) | | 0.00(0.00-0.25) | | 0.00(0.00-0.22) | | | 0.00(0.00-0.17) | | <.001 |
| Reflux | 0.00(0.00-0.00) | | 0.00(0.00-0.00) | | 0.00(0.00-0.00) | | | 0.00(0.00-0.00) | | <.001 |
| Anxiety | 0.00(0.00-0.00) | | 0.00(0.00-0.22) | | 0.00(0.00-0.20) | | | 0.00(0.00-0.29) | | <.001 |
| Depression | 0.00(0.00-0.00) | | 0.00(0.00-0.00) | | 0.00(0.00-0.00) | | | 0.00(0.00-0.04) | | <.001 |
| Delirium | 0.00(0.00-0.00) | | 0.00(0.00-0.00) | | 0.00(0.00-0.00) | | | 0.00(0.00-0.00) | | <.001 |

**Table S3 Mediation Analyses of Symptoms in Improving Energy/Protein Intake Via ePROM**

| Exposure | Mediators | Adequate Energy Intake | | | Adequate Protein Intake | | |
| --- | --- | --- | --- | --- | --- | --- | --- |
|  |  | Indirect Effects | Proportion Mediated | P value | Indirect Effects | Proportion Mediated | P value |
| Adherence Duration for EPRO  Journal | Pain | 0.00(-0.00 to 0.00) | 0.01(-0.00 to 0.02) | 0.160 | 0.00(-0.00 to 0.00) | 0.00(-0.00 to 0.01) | 0.060 |
|  | Fatigue | -0.00(-0.00 to 0.00) | -0.02(-0.06 to 0.01) | 0.200 | -0.00(-0.00 to 0.00) | -0.01(-0.02 to 0.00) | 0.190 |
|  | Dyspnea | -0.00(-0.00 to 0.00) | -0.00(-0.00 to 0.01) | 0.580 | -0.00(-0.00 to 0.00) | -0.01(-0.02 to 0.00) | 0.240 |
|  | Cough | 0.00(-0.00 to 0.00) | 0.01(-0.01 to 0.02) | 0.790 | -0.00(-0.00 to 0.00) | -0.00(-0.01 to 0.00) | 0.750 |
|  | Appetite | -0.00(-0.00 to 0.00) | -0.01(-0.03 to 0.00) | 0.130 | -0.00(-0.00 to 0.00) | -0.00(-0.01 to 0.00) | 0.170 |
|  | Dry Mouth | 0.00(-0.00 to 0.00) | 0.00(-0.00 to 0.02) | 0.400 | 0.00(-0.00 to 0.00) | 0.01(-0.00 to 0.01) | 0.058 |
|  | Nausea | 0.00(-0.00 to 0.00) | 0.01(-0.02 to 0.04) | 0.400 | 0.00(-0.00 to 0.00) | 0.01(-0.01 to 0.02) | 0.360 |
|  | Bloating | 0.00(-0.00 to 0.00) | 0.00(-0.00-0.01) | 0.910 | 0.00(-0.00 to 0.00) | 0.00(-0.00 to 0.01_) | 0.380 |
|  | Diarrhea | -0.00(-0.00 to 0.00) | -0.00(-0.02 to 0.01) | 0.800 | -0.00(-0.00 to 0.00) | -0.00(-0.01 to 0.00) | 0.270 |
|  | Constipation | 0.00(0.00 to 0.00) | 0.01(0.00 to 0.03) | **0.034** | 0.00(0.00 to 0.00) | 0.01(0.00 to 0.02) | **0.002** |
|  | Reflux | -0.00(-0.00 to 0.00) | -0.02(-0.10 to 0.03) | 0.480 | -0.00(-0.00 to 0.00) | -0.04(-0.10 to -0.01) | **0.002** |
|  | Anxiety | -0.00(-0.00 to 0.00) | -0.00(-0.02 to 0.01) | 0.530 | 0.00(-0.00 to 0.00) | 0.00(-0.00 to 0.00) | 0.610 |
|  | Depression | 0.00(-0.00 to 0.00) | 0.00(-0.00 to 0.01) | 0.850 | -0.00(-0.00 to 0.00) | -0.00(-0.00 to 0.00) | 0.320 |
|  | Delirium | -0.00(-0.00 to 0.00) | -0.00(-0.01 to 0.01) | 0.980 | -0.00(-0.00 to 0.00) | -0.00(-0.01 to 0.00) | **0.006** |
